# Heart rate variability after radiofrequency ablation of epicardial ganglionated plexuses on the ovine left atrium

**DOI:** 10.1186/s12872-017-0727-7

**Published:** 2017-12-12

**Authors:** Vilius Kviesulaitis, Aras Puodziukynas, Dainius Haroldas Pauza, Vytautas Zabiela, Tomas Kazakevicius, Raimundas Vaitkevicius, Evaldas Diržinauskas, Vytenis Semaška, Antanas Strazdas, Ruta Unikaite, Kristina Rysevaite, Neringa Pauziene, Remigijus Zaliunas

**Affiliations:** Lithuanian University of Health Sciences, Eivenių 2, LT-50161 Kaunas, Lithuania

**Keywords:** Radio frequency ablation, Intrinsic cardiac nerves, Ganglionated plexuses, Left atrium, Ovine, Heart rate variability

## Abstract

**Background:**

Ganglionated plexuses (GP) are terminal parts of cardiac autonomous nervous system (ANS). Radiofrequency ablation (RFA) for atrial fibrillation (AF) possibly affects GP. Changes in heart rate variability (HRV) after RFA can reflect ANS modulation.

**Methods:**

Epicardial RFA of GP on the left atrium (LA) was performed under the general anesthesia in 15 mature Romanov sheep. HRV was used to assess the alterations in autonomic regulation of the heart. A 24 − hour ECG monitoring was performed before the ablation, 2 days after it and at each of the 12 following months. Ablation sites were evaluated histologically.

**Results:**

There was an instant change in HRV parameters after the ablation. A standard deviation of all intervals between normal QRS (SDNN), a square root of the mean of the squared differences between successive normal QRS intervals (RMSSD) along with HRV triangular index (TI), low frequency (LF) power and high frequency (HF) power decreased, while LF/HF ratio increased. Both the SDNN, LF power and the HF power changes persisted throughout the 12 − month follow − up. Significant decrease in RMSSD persisted only for 3 months, HRV TI for 6 months and increase in LF/HF ratio for 7 months of the follow − up. Afterwards these three parameters were not different from the preprocedural values.

**Conclusions:**

Epicardial RFA of GP’s on the ovine left atrium has lasting effect on the main HRV parameters (SDNN, HF power and LF power). The normalization of RMSSD, HRV TI and LF/HF suggests that HRV after epicardial RFA of GPs on the left atrium might restore over time.

## Background

Autonomic nervous system regulates the physiological processes of the heart [1]. It also plays a part in the pathogenesis of AF [2]. Pulmonary vein (PV) root isolation is a routine procedure to treat AF. GP and neural pathways are affected inevitably during the procedure as they anatomically overlap the sites of ablation. The purpose of the present study was to determine the long term alterations in HRV after the RFA of GP on the LA. Determined to avoid ethical limitations and medical conditions common in the human studies, we chose a sheep model which heart is well examined neuroanatomically and is rather similar in size to human [3].

## Methods

The present study was conducted under the permission (No.: 33ĮV-62) issued by the State Food and Veterinary Service, complying to DIRECTIVE 2010/63/EU. Fifteen mature (6–8 months old) Romanov sheep of either gender were acquired in a local farm, where they spent most of the study period in the maternal herd under the supervision of original owner and veterinary doctor, except during the perioperative period when they were tended in veterinary clinics.

Left thoracotomy was performed under the general anesthesia following standardized protocol from our previous study [4]: 0.1 mg/kg Xylazine was administered i.m. for sedation, and anesthesia initiated with 0.03 mg/kg of Midazolam i.m. and 2 mg/kg of Ketamine i.m.. After intubation anesthesia was maintained with intravenous 5 μg/kg Phentanyl and ventilation with Halothane through a cyclic breathing system. Infusion of Ringer’s solution was administered 15 mL/kg/h during the anesthesia. Pericardial space was opened and dorsal LA area revealed. An extensive RFA was performed covering area encircled by left and middle pulmonary, left azygos, coronary sinus and caudal vein. Left atrium dorsal and middle dorsal epicardial ganglionated subplexuses are located in this area. Together they contain about 40% of total epicardial ganglia of the ovine heart [3]. Standard 4 mm irrigated tip ablation catheters (*Biosense Webster, Thermocool, Diamond Bar*) irrigated with the isotonic solution were used. Ten to fifteen RF energy applications of 20 W power, 500 kHz frequency were applied for 30 s under direct visual control with the +50 °C tissue temperature safety cutout. “Popping” phenomenon was avoided. Perioperative medication did not include any long − term neuroactive substances except those used for the anesthesia and antibiotic therapy. Thirteen sheep have survived. One animal died due to injury of the left coronary artery and resulting myocardial infarction immediately after the procedure, the second died due to pneumonia 2 months later. Remaining sheep were euthanized 1 year after the RFA.

All sheep underwent a 24 h ECG monitoring the day before the ablation. On the second day after the ablation a 24 h ECG was recorded again and repeated every month throughout a 12 − month period. *SR-Medizinetechnik Cardioscout recorder* hardware was used for the ECG monitoring. *Cardio Explorer version 3.2* software was used to process the monitoring data and artefacts were corrected manually. *Kubios HRV version 2.1* software was used to evaluate time, frequency and geometrical domain parameters of HRV. For the time domain we have chosen SDNN, RMSSD and a percentage of differences between successive normal to normal QRS intervals greater than 50 ms (pNN50). For the frequency domain we have chosen: very low frequency (VLF) band power (band range from 0.0033 to 0.04 Hz), LF band power (band range from 0.04 to 0.15 Hz), HF band power (band range from 0.15 to 0.4 Hz) and LF/HF ratio. HRV TI – an integral of the density distribution (count of all normal to normal QRS intervals) divided by the maximum of the density distribution was chosen as a geometrical method.

Histochemical evaluation of LA for acetylcholinesterase (AChE) was performed to determine whether the general structural organization of the left dorsal neural subplexus is altered by RFA after 12 months. Sheep hearts from control (no intervention) and experimental groups were perfused with cold phosphate buffered saline (pH 7.4) via coronary vessels and prefixed for 15 min at room temperature in 4% paraformaldehyde solution. The region of the left dorsal atrial subplexus on the coronary sinus was extirpated as it shown in the picture 1a. The extirpated tissue sample was stained histochemically for AChE [3]. Neural structures stained for AChE were examined and photographed using a stereo microscope Stemi 2000 CS (Carl Zeiss, Jena, Germany) applying a fiber optic light illuminator KL 2500 LCD (Schot AG, Mainz, Germany). Images were captured using a digital camera Axiocam MRc5 (Zeiss, Gottingen, Germany).

Immunohistochemistry for intrinsic neural structures was performed on tissue cryosections [3] using polyclonal primary mouse monoclonal anti-protein gene product 9.5 (PGP 9.5; dilution 1:1000; ab8189, Abcam, Cambridge, UK.) and polyclonal rabbit anti-tyrosine hydroxylase (TH, dilution 1:1000, AB152, Chemicon International, Temecula, California, USA). The applied species specific secondary antibodies were conjugated, respectively, to Alexa Fluor 488 (dilution 1:500, A21202, Invitrogen Corp., Carlsbad, CA, USA) and Cy3 (AP182C, Chemicon International, Temecula, California, USA). The immunohistochemically stained sections were analyzed and photographed using a confocal laser scanning microscope LSM 700 with the software package ZEN 2010 (Carl Zeiss, Jena, Germany).

Statistical data were processed using *SPSS 21.0* statistical package. Mean values of above − mentioned HRV components were calculated. Friedman and Wilcoxon nonparametric criteria were used to analyze the quantitative data. Data were considered significant at *p* < 0.05.

## Results

### Changes in HRV

**Fig. 1:**
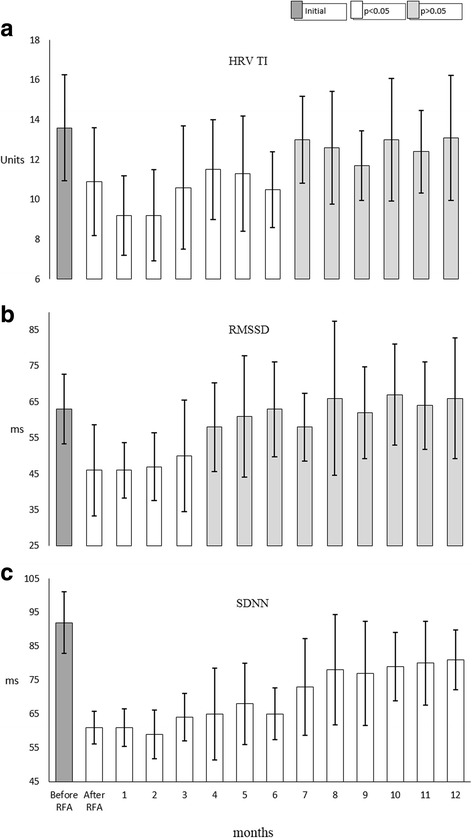
Changes in time and geometrical domain parameters of heart rate variability during a 12 − month follow − up.A sharp decrease in medium HRV geometrical and time domain parameter values were notable in 13 sheep immediately after the RFA in the left atrium. HRV TI (**a**) remained decreased for 6 months and RMSSD (**b**) for 3 months before returning to preprocedural values. SDNN (**c**) remained decreased for a whole study period, though with a tendency to increase towards the end of the 12 months follow – up

We did not find any statistically significant variations in heart rate of experimental animals. But significant changes in HRV parameters were notable immediately after the RFA. Time domain parameters have decreased immediately after the ablation (Fig. 1): mean SDNN before the RFA was 92.15 ± 9.13 ms and decreased to 60.41 ± 4.88 ms (*p* < 0.05), RMSSD was 62.63 ± 9.64 ms before and 44.30 ± 12.71 ms (*p* < 0.05) after the ablation. The decrease of mean pNN50 from 25.08 ± 10.49% to 21.98 ± 8.08% (*p* = 0.475) was statistically insignificant due to overall dispersion throughout the 12 − month follow − up. HRV TI significantly decreased from 13.51 ± 2.67 to 10.55 ± 2.69 (*p* < 0.05) after the RFA.

Initial changes of HRV TI, SDNN and RMSSD persisted for different periods of the follow − up (Fig. 1a, b and c). Decrease of SDNN persisted for the whole period of the study. RMSSD returned to the values which were not significantly different from the preprocedural within 3 months after the RFA. HRV TI behaved similarly within 6 months of the follow − up. Both RMSSD and HRV TI remained stable after the normalization.

**Fig. 2:**
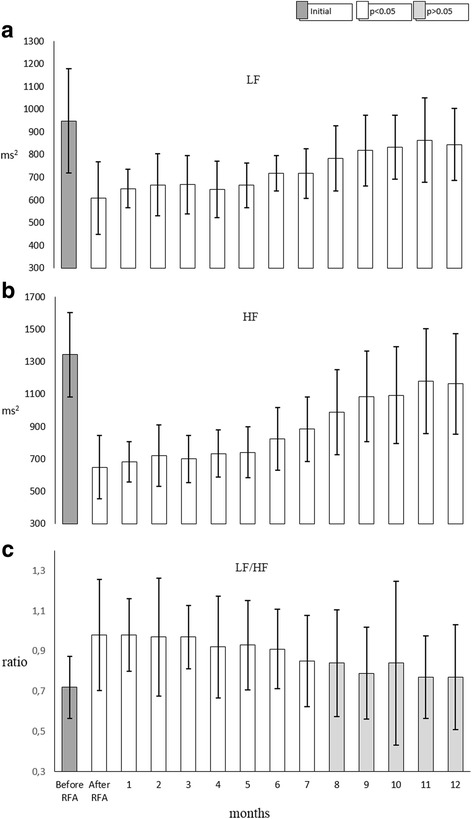
Changes in frequency domain parameters during a 12 − month follow − up. Both medium LF (**a**) and HF (**b**) components of HRV frequency domain of 13 study animals decreased immediately after the RFA in the left atrium. These changes persisted through the follow – up of 12 months. LF/HF ratio (**c**) increased after the procedure but returned to preprocedural values after 7 months

Frequency domain parameters were also affected by the ablation. LF and HF powers decreased significantly comparing to preprocedural values (Fig. 2a, b ). LF component has decreased from 987 ± 229 ms^2^ to 603 ± 159 ms^2^ (*p* < 0.005) and HF power - from 1386 ± 259 ms^2^ to 653 ± 193 ms^2^ (*p* < 0.05). The extent of decrease of both parameters was not proportional leading to the increase of LF/HF ratio (Fig. 2c ) from 0.726 ± 0.155 to 0.963 ± 0.277 (*p* < 0.05). Both LF and HF components remained decreased during the whole study period, but LF/HF ratio gradually decreased and in 7 months there was no statistically significant difference comparing to preprocedural values. Though initially after the ablation VLF power showed a numerical decrease from 2957 ± 1855 ms^2^ to 2285 ± 1846 ms^2^, no statistically significant change was observed comparing monthly results with preprocedural values through the study period.

### Neurohistological observations

**Fig. 3:**
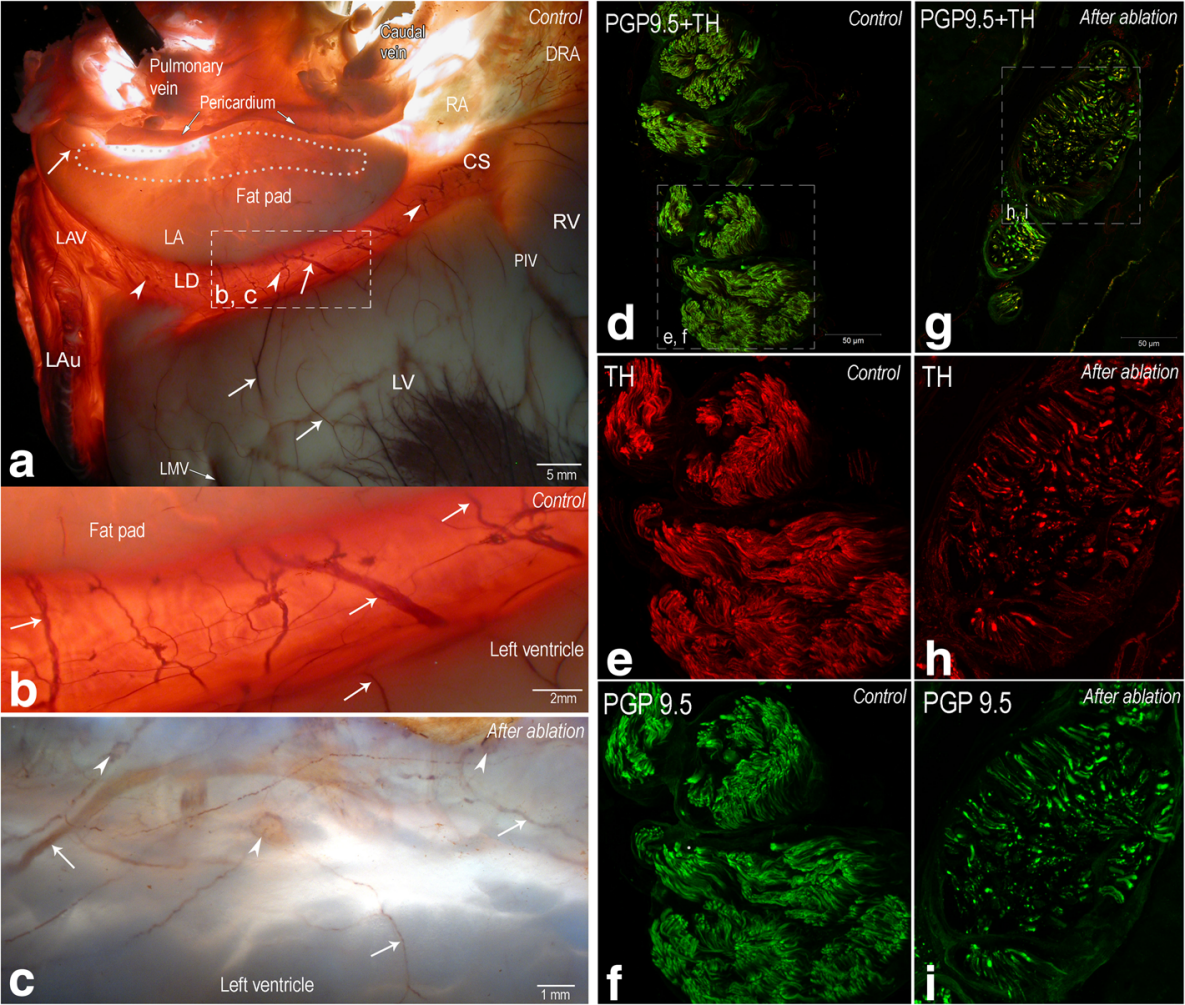
Histochemical findings after radiofrequency ablation in left atrium. **a** Left dorsal view of pressure-inflated sheep heart stained for AChE to demonstrate the epicardial nerves (white arrows) and ganglia (white arrowheads) on the dorsal left atrium, coronary sinus, and on the left dorsal ventricle. In the panel (**b**), the enlarged area boxed in the panel (**a**) illustrates the epicardial neural structures that were sampled and examined applying immunohistochemical method. **c** The dorsal left atrial area topographically identical to the boxed area in panel (**a**) 12 months after the epicardial RFA at the root of left and middle pulmonary veins (shown as dashed area on panel **a**). Note the epicardial nerves (white arrows) and ganglia (white arrowheads) that are evidently less histochemically positive for AChE following RFA at the roots of pulmonary veins. Panels (**d-i**) illustrate the transverse sections of the epicardial nerves that were immunohistochemically stained for PGP 9.5 (green color) and TH (red color) in control samples (**d**-**f**) and one year after RFA (**g**-**i**). Note the evidently lesser density of nerve fibers (positive for both the PGP9.5 and TH) in the experimental group nerves compared to the controls (panels e-f vs. **h**-**i**)

Histological AChE staining demonstrates that majority of epicardial nerves and ganglia in the left dorsal neural subplexus were detectable in both the control and the ablated hearts (Fig. 3a-c). However, the epicardial nerves and ganglia on the dorsal left ventricle and coronary sinus from the control animal group were evidently sharper stained for AChE in comparison to the ablated hearts as staining of intrinsic neural structures of the latter hearts was manifestly obscure at the place below the RFA sites (Fig. 3a-b vs. Fig. 3c ).

Immunohistochemistry for general neural marker protein gene product 9.5 (PGP 9.5) and adrenergic marker to tyrosine hydroxylase (TH) demonstrates staining both in the control and experimental animal groups. Control epicardial nerves from the left dorsal neural subplexus of all examined animals were densely and abundantly packed by nerve fibers that were evenly distributed in the transverse sections of the nerves (Fig. 3d-f). However, immunohistochemistry performed on the transverse nerve sections extirpated from the dorsal left atrial walls of the experimental animals demonstrates a sharp decrease in number and the patchy distribution of axons positive for both the PGP9.5 and TH (Fig. 3g-i). These findings confirm that neural structures were affected by RFA of the dorsal left atrial neural structures in the group of experimental animals.

## Discussion

Evidence of ANS being involved in the pathogenesis of AF naturally suggests many therapeutic insights and clinical applications. Straightforward ablation of GP is not yet accepted as a routine therapeutic tool for AF treatment. On the other hand, it should not be considered as purely experimental. Pulmonary vein isolation for the treatment of AF is an accepted routine procedure already. Because of the anatomical overlapping of the structures it also affects the GP densely situated around PV roots. And functional modulation of cardiac ANS is notable after the PV isolation. Thus, PV isolation can be considered as a GP ablation of limited extent.

A considerable number of studies have analyzed modification of ANS after PV isolation. HRV is a handy noninvasive tool universally used for this purpose. Therefore, we have accepted it as a method of ANS evaluation in our study. Frequency and time domain components of the HRV reflect the fluctuations of the autonomic input to the heart. HF band power specifically represents the vagal tone as LF bands represent both vagal and sympathetic influence. Ratio between LF/HF mimics the autonomic balance between the vagal and sympathetic tones. It is important to acknowledge that HRV reflects the fluctuations of ANS without a quantitative ability [5, 6] and we could not speculate which component of ANS was modulated more in our study. On the other hand, ganglionated plexuses in the left atrium are of very intricate composition of sympathetic, parasympathetic and mixed nerves [2]. This makes the selective ablation almost impossible. So isolated effect on one of the systems cannot be expected. We could interpret that according to the increase in LF/HF ratio ablation affected more sympathetic nerve fibers. Though, as both frequencies decreased significantly it would be difficult to prove such a fact.

PV ablation procedures for paroxysmal AF in humans demonstrate an acute universal adjustment of HRV time domain parameters. Usually SDNN, RMSSD and pNN50 significantly decreases after the procedure [7–15]. Moreover, successful procedures (without recurrence of AF) have shown stronger effects of ablation on SDNN and RMSSD [7, 8, 10] compared to those with AF recurrence. This might reflect the extent and precision of the ablation on intracardiac structures of ANS. While we performed ablation on ovine heart, our results had similar trends as in human studies. It might suggest that autonomous modulation works similarly in both species. Thus, regarding autonomic innervation, ovine heart can be considered as a study object. Moreover, experimental approaches like histological evaluation otherwise inappropriate in human studies make this method particularly valuable. We recognize the limitation of our study as we did not control for possible acute effects of thoracotomy and pericardial sack opening on the HRV. But as thoracic and pericardial nerves have no direct interconnections with intracardiac nerves we did not expect a strong HRV response to the procedure.

There is a degree of disparity noted in studies concerning the changes in frequency domain parameters of the HRV after the PV isolation. Studies demonstrate significant decrease in LF and HF powers immediately after the procedure [7, 8, 12, 14, 16–19], which corresponds with our findings. Although changes in LF/HF ratio are more contradictory. As presented in different studies LF/HF ratio has either increased [12, 15, 18], did not change [14, 17, 19] or decreased [7, 8, 17]. Those data might discredit the LF/HF ratio as a proper parameter for evaluation of ANS tone as there were no obvious differences between the study populations. Different techniques of PV isolation (segmental and circumferential approach) might had impact on that. However, K. Wang et al. [8] did not find any significant difference between these two techniques on ANS tone.

More GP ablation − focused studies when compared to PV isolation alone have demonstrated similar results. Kang KW with colleagues enhanced circumferential PV isolation adding additional lesion connecting highest septal aspect of superior v. cava with right atrial septum for additional effect on ANS [20]. This approach significantly decreased both time and frequency components of HRV compared to the circumferential PV isolation alone. C. Pappone has studied a group of patients after circumferential PV isolation with additional extensive vagal denervation procedure [21]. Additional increase of LF/HF along with usual effects of PV ablation on HRV was noted in this study. Some human studies of isolated GP ablation showed post procedural decrease in both HF and LF powers [22–24]. E. Pokushalov repeatedly confirmed increased LF and decreased HF powers along with increase in LF/HF ratio after GP ablation [25, 26]. Our study has also presented similar results. It shows that destruction of GP extensively modulates the autonomic regulation of the heart. Usually greater ANS modification expressed by the changes in HRV is noted in patients without AF recurrence. This observation complements the theory of ANS involvement in pathogenesis of AF and gives reason for further research.

Regardless the evident acute changes in cardiac autonomic regulation after the ablation, not all the effects seem to be permanent, which was observed in our study too. Existing data on the duration of changes of autonomic regulation are diverse. L. Callo presented data from the 19.7 ± 5.2 months’ follow − up of the patients after the GP ablation [24]. Significant modification of HRV in patients without AF recurrence lasting up to 6 months was observed. Unfortunately, no data on further changes are available, thought the follow-up was significantly longer. In patients with AF recurrence, HRV returned to initial values earlier during the period between 3 and 6 months. E. Pokushalov also observed the normalization of HRV changes in 6 months after GP ablation [25]. C. Pappone. has observed significant HRV changes up to 3 months after extensive vagal denervation along with circumferential PV isolation [21]. Ablation of parasympathetic vagal structures did not prolong the effect of PV isolation on the duration of the changes in HRV. Some other PV isolation studies demonstrate modification of ANS tone lasting up to 12 months after the procedure, unfortunately no longer-term follow-ups were documented [14, 18, 19]. Other studies presented even shorter follow − up periods of 3 to 6 months [7, 12, 17, 20].

It is difficult to point out the possible mechanisms of ANS activity normalization after the ablation and the different duration of this process. It remains unclear whether the normalization is a natural process or a flaw in the therapy. Properties of the study population, medications and different follow − up strategies probably have a solid impact on the outcome. It is probable that parts of ANS unaffected by the ablation are partially responsible for the normalization of some HRV parameters. Also, there are different opinions about the possibility of ANS reinnervation. Q. Y. Zhao has designed an animal study on dogs showing evidence of neural remodeling after GP ablation in the right atrium [27]. Another study on reablation for AF recurrence confirmed the absence of high frequency stimulation − induced vagal reflexes [28]. These data contradict the reinnervation. On the other hand, it could be explained by a relatively short mean period between the procedures, which was about 4 months. While it is unclear how long would it take for a hypothetical reinnervation of the heart, most studies observe normalization of ANS parameters in 6 months. Our previous study [4] on the ovine heart after GP ablation also demonstrated no histological evidence of neural regeneration. Though examination was also performed only 2–3 months after the ablation, the evidence of absent regeneration was strong. Further experiments on animal models might give greater insight into this problem.

## Conclusions

After the epicardial RFA of GP on the ovine LA an immediate modification of geometrical, time and frequency domains of the HRV has occurred. Changes of SDNN, HF power and LF power persisted for 12 months’ follow − up and may be considered as possible criteria of efficacy of AF ablation in a clinical practice. On the other hand, changes in parameters like RMSSD, HRV TI and LF/HF ratio were less persistent. They returned to values that were not significantly different comparing to preprocedural in 3–7 months. This might hint of ANS of the heart regaining its functions over time.

## Abbreviations

AChE: Acetylcholinesterase
AF: Atrial fibrillation
ANS: Cardiac autonomous nervous system
GP: Ganglionated plexuses
HF: High frequency
HRV: Heart rate variability
LA: Left atrium
LF: Low frequency
PGP 9.5: Protein gene product 9.5
pNN50: A percentage of differences between successive normal to normal QRS intervals greater than 50 ms
PV: Pulmonary vein
RFA: Radiofrequency ablation
RMSSD: A square root of the mean of the squared differences between successive normal QRS intervals
SDNN: A standard deviation of all intervals between normal QRS
TH: Tyrosine hydroxylase
TI: Triangular index
VLF: Very low frequency
